# Advances and Challenges in Microbial Immobilization Technology for Organic Contaminated Soil Remediation

**DOI:** 10.3390/toxics14010003

**Published:** 2025-12-19

**Authors:** Kai Yao, Yaqiong Wang, Mohan Bai, Xiaodong Ma, Huike Ye

**Affiliations:** 1 School of Ecology, Environment and Resources, Qinghai Minzu University, Xining 810007, Chinamaxiaodongthu@foxmail.com (X.M.); 2 Agro-Environmental Protection Institute, Ministry of Agriculture and Rural Affairs, Key Laboratory of Original Agro-Environmental Pollution Prevention and Control, MARA, Tianjin Key Laboratory of Agro-Environment and Agro-Product Safety, Tianjin 300191, China; baimohan@caas.cn; 3 Qinghai Provincial Biotechnology and Analytical Test Key Laboratory, Xining 810007, China; 4 Qinghai Provincial Key Laboratory of High-Value Utilization of Characteristic Economic Plants, Xining 810007, China

**Keywords:** microbial immobilization, organic pollutants, soil remediation, biodegradation, immobilized carrier

## Abstract

Organic pollutants, representing a major category of soil contamination, not only significantly impair soil environmental quality but also threaten human health and agricultural safety through biological accumulation in the food chain. Microbial immobilization technology, as a sustainable and efficient remediation strategy, provides a promising solution for mitigating soil organic pollution. By immobilizing functional microorganisms on specific carriers, this technology effectively shields microorganisms from environmental stressors, extends their active lifespan, and markedly improves microbial stability and pollutant degradation efficiency. Nevertheless, despite its substantial potential, the large-scale application of microbial immobilization technology encounters several challenges, including the optimization of carrier materials, enhancement of microbial community stability, and mitigation of environmental impacts. This paper comprehensively reviews the advancements and challenges of microbial immobilization technology in the remediation of organically contaminated soils. It emphasizes that future research should prioritize the development of cost-effective, high-performance carriers, the refinement of immobilization processes, and the exploration of synergistic interactions within microbial communities to achieve efficient and eco-friendly soil remediation objectives. To advance this field, future efforts must bridge fundamental research on carrier–microbe interactions with engineering-scale validation, addressing key challenges in cost, stability, and predictability for field applications.

## 1. Introduction

Over the years, soil environmental pollution caused by improper disposal of chemical waste has become increasingly prominent with the acceleration of industrialization [1]. Research indicates that the extensive economic growth model and imbalanced industrial structure layout prevalent worldwide, particularly the substantial waste emissions generated during rapid industrial development stages, have exerted profound impacts on soil environments [2]. Currently, soil pollution has evolved into a global environmental issue, exhibiting significant regional characteristics in its spatial distribution. Among various pollutants, organic contaminants, as a primary category of soil pollution, not only significantly degrade soil environmental quality but also potentially accumulate through biological magnification in the food chain, posing substantial threats to agricultural product safety and human health [3]. Studies have demonstrated that the persistent accumulation of organic pollutants in soil can alter soil physicochemical properties, disrupt soil microbial community structures, and consequently affect soil ecological functions and normal crop growth [4]. More critically, these pollutants can enter the human body through multiple exposure pathways such as “soil–plant-human” or “soil-water-human” routes, where long-term low-dose exposure may lead to chronic poisoning and various health risks. In light of these concerns, research on the remediation and restoration of organically contaminated soils has become an urgent and critical issue in the environmental field [5].

Organic pollutants pose a significant threat to ecosystems and human health. Microbial remediation has emerged as a promising solution due to its environmental friendliness, cost-effectiveness, and high degradation efficiency [6]. Microorganisms can degrade or remove pollutants through metabolic activities, adsorption, migration, and transformation [7]. However, free microbial cells in natural environments are susceptible to abiotic factors (e.g., temperature, oxygen, and moisture) and biotic competition from indigenous microorganisms, leading to reduced survival rates and metabolic activity, thereby limiting their remediation efficiency [8,9]. To address these challenges, microbial immobilization technology has been developed. This technique involves fixing free microbial cells onto carrier materials via physical or chemical methods, enhancing their environmental adaptability, stability, and degradation performance [10]. The key factors in microbial immobilization are the selection of immobilization methods and carrier materials [10,11]. Additionally, nutrients such as organic matter, nitrogen, phosphorus, and potassium in the carrier can support microbial growth [12], while surface functional groups (e.g., carboxyl and hydroxyl groups) may further enhance pollutant degradation through catalytic oxidation [13].

This review systematically summarizes recent advances in the application of immobilized microbial consortia for organic-contaminated soil remediation, with a focus on optimization strategies for different immobilization methods and carrier materials. The findings aim to provide theoretical and practical insights for future developments in soil remediation technologies.

## 2. Overview of Organic Contaminated Soil Remediation Technologies

### 2.1. Physical Remediation Technologies

Physical remediation technologies achieve pollutant separation or concentration reduction through physical methods, including soil replacement, vitrification, thermal treatment, electro-thermal desorption, thermal conduction heating, and steam-enhanced extraction [14]. These technologies are suitable for severely contaminated soils with limited spatial extent and can rapidly reduce pollutant concentrations. However, their limitations include high implementation costs and the inability to completely remove pollutants, often resulting in pollutant transfer and potential secondary pollution [15]. For example, soil replacement can quickly remove contaminated soil but requires significant labor, material, and transportation costs, and the subsequent treatment of contaminated soil remains a challenge [16]. Thermal treatment and vitrification effectively reduce pollutant concentrations, but their high energy consumption and costs limit their large-scale application [17]. Therefore, physical remediation technologies offer rapid results for heavily contaminated soils, but their high costs and pollutant transfer issues limit their widespread application (Table 1).

### 2.2. Chemical Remediation Technologies

Chemical remediation technologies introduce specific chemical reagents (e.g., oxidants, reductants, or stabilizers) into the soil to induce chemical reactions that transform pollutants into less toxic or harmless substances [21]. Common chemical remediation methods include steam extraction, redox reactions, Fenton’s reagent oxidation, ozone oxidation, plasma degradation, photocatalytic degradation, and incineration [22,23]. Although chemical remediation significantly enhances remediation efficiency, it may introduce new chemicals, disrupt the soil’s ecological balance, and pose environmental risks. For instance, Fenton’s reagent oxidation efficiently degrades organic pollutants but generates iron sludge and acidic conditions that may negatively impact soil ecosystems [24]. Incineration completely removes pollutants but faces challenges related to high energy consumption and secondary pollution. Therefore, chemical remediation technologies excel in improving remediation efficiency, but their potential for secondary pollution and ecological disruption requires attention.

### 2.3. Biological Remediation Technologies

Bioremediation is a green remediation method that utilizes the metabolic processes of microorganisms, plants, or animals to transform soil pollutants into less toxic or harmless substances [5,25]. This technology primarily includes phytoremediation [26], soil fauna remediation [27], and microbial remediation [28], with microbial remediation being the most widely used due to its efficiency and broad applicability [29,30]. Microorganisms utilize organic pollutants in the soil as carbon sources, degrading them into CO_2_, water, or simple small-molecule alcohols and acids through their growth, reproduction, and metabolic activities, thereby achieving soil remediation goals [31,32]. Among the various microorganisms capable of degrading organic pollutants, bacteria (e.g., *Pseudomonas adaceae*, *Bacillus*, *Flavobacterium*, *Alcaligenes*, *Acinetobacter*, *Rhodococcus equi*, and *Corynebacterium*) [33], fungi (e.g., *Aspergillus*, *Penicillium*, *Rhizopus*, *Trichoderma*, whiterot fungi, and *Mucor*) [34], and actinomycetes (e.g., *Nocardia* and *Streptomyces*) play significant roles [35]. Notably, the genus *Pseudomonas* has emerged as one of the most representative microbial species in this field due to its exceptional ability to degrade various organic pollutants, including pesticides and aromatic hydrocarbons [36]. Bioremediation technologies, with their environmental friendliness and cost-effectiveness, have become a mainstream approach in soil remediation.

### 2.4. Advances and Applications of Bioremediation Technologies

Since the 1980s, bioremediation technologies have achieved significant progress. These technologies rely on natural microorganisms in soil or artificially selected functional microbial communities to degrade, transform, or immobilize pollutants through their metabolic activities, thereby achieving soil remediation goals [37,38]. Bioremediation technologies are characterized by simple equipment, flexible operation, low cost, and high efficiency, making them a key research direction in soil remediation [20]. With advancements in modern biotechnology, microbial remediation techniques have been further optimized. Researchers have screened high-efficiency degrading strains, optimized microbial community combinations, and applied them to diverse soil pollution remediation scenarios [39,40]. Practical applications have demonstrated that microbial remediation not only offers high treatment efficiency and environmental friendliness but also minimizes disturbance to soil structure. In microbial remediation technologies, stimulating and enhancing the degradation capabilities of indigenous microorganisms is a commonly used approach. For example, biological ventilation technology accelerates the bioremediation process of organic pollutants by delivering oxygen to unsaturated soil zones, thereby activating indigenous microorganisms [41]. This technique is particularly suitable for in situ remediation of sites contaminated with light petroleum. However, it has specific requirements for soil moisture content and temperature, and its treatment costs are influenced by factors such as soil type and area. Biojet technology enhances microbial activity by injecting air into subsurface soils, promoting pollutant removal [42]. This method facilitates the migration of volatile organic compounds from saturated to unsaturated zones and is widely used for diesel, kerosene-contaminated aquifers, or soils, with applicability extending to other volatile pollutants [43].

Furthermore, bio-stimulation technology initiates the bioremediation process by adding nutrients (e.g., phosphorus, nitrogen, oxygen, carbon, and electron acceptors) to promote the growth of native bacteria in contaminated sites [44]. Under anaerobic conditions, the addition of electron donors can induce the remediation of halogenated pollutants by enabling microorganisms to accept their electrons. Unlike bio-stimulation, bioaugmentation involves the introduction of exogenous microorganisms to accelerate pollutant degradation, while bio-stimulation enhances the metabolic activity of native bacteria through nutrient supplementation [40,45]. Studies have shown that the application of bio-stimulation technology in contaminated areas can significantly improve the degradation efficiency of indigenous microorganisms.

In summary, organic contaminated soil remediation technologies encompass three major categories: physical, chemical, and biological remediation, each with its unique advantages and limitations [46]. Physical remediation technologies are suitable for rapidly reducing pollutant concentrations but face challenges such as high costs and pollutant transfer issues [18]. Chemical remediation technologies offer high efficiency but may introduce secondary pollution [19]. Bioremediation technologies, characterized by their environmental friendliness and cost-effectiveness, have become the mainstream approach in soil remediation [20]. With advancements in modern biotechnology, microbial remediation technologies have significantly improved remediation efficiency through optimized microbial community combinations and enhanced indigenous microbial functions [47,48]. Given the diversity of pollutants in soils and the frequent cooccurrence of organic and inorganic contaminants, selecting an appropriate remediation method is challenging, especially when balancing environmental sustainability and treatment efficiency. Therefore, in practical applications, it is essential to comprehensively consider site characteristics, contamination levels, development plans, and treatment costs, while adhering to core principles such as technological maturity, target pollutant compatibility, soil type suitability, cost-effectiveness, contaminant removal rate, remediation duration, and prevention of secondary pollution to achieve optimal remediation outcomes [49]. In summary, while bioremediation presents a sustainable alternative to physical and chemical methods, the limited environmental resilience and low survival rates of free microbial cells hinder their efficacy. This limitation underscores the necessity for advanced strategies to protect and enhance microbial functionality in situ, leading to the development of microbial immobilization technology as a pivotal advancement, which will be detailed in the following sections.

## 3. Application of Microbial Immobilization Technology

Traditional free microbial agents face numerous challenges in the field of bioremediation of soils, including weak environmental tolerance, susceptibility to competitive inhibition by indigenous soil microorganisms, and short activity duration, which significantly limit their widespread application, so to address these limitations, immobilization technology, which emerged in the 1980s, has been introduced into bioremediation of soils [50]. This technology immobilizes functional microorganisms within specific microenvironments, effectively increasing microbial density and activity while significantly enhancing their tolerance and stability against adverse environmental factors (such as pH, temperature, and toxic substances), thereby substantially improving bioremediation efficiency [10,11]. Research has demonstrated that immobilization technology not only extends the active lifespan of functional microorganisms but also reduces microbial loss, providing more reliable technical support for soil pollution remediation. Furthermore, immobilization technology exhibits promising application prospects, offering a novel and effective approach to addressing complex soil contamination issues [51].

### 3.1. Methods of Microbial Immobilization Technology

Currently, microbial immobilization technologies primarily include adsorption [52], embedding [53], cross-linking [54], and covalent binding [55] (Table 2). Cross-linking typically involves using bifunctional reagents (e.g., glutaraldehyde, genipin) to form stable networks between microbial cell surfaces or between cells and carrier functional groups [54]. Covalent binding directly attaches microbial cells to activated carriers (e.g., silane-modified surfaces, carboxylated polymers) via stable covalent bonds, often resulting in lower cell leakage but requiring careful control of reaction conditions to maintain cell viability [55]. These methods offer enhanced stability compared to physical adsorption but may involve chemicals that require post-treatment. Among these, adsorption and entrapment methods have become the most widely studied and applied due to their unique advantages [10].

The adsorption method relies on electrostatic attraction and physical adhesion to immobilize microbial cells within porous carrier materials [52]. This approach offers advantages such as simple operation, minimal impact on microbial cell activity, and recyclability of the carrier materials. However, the adsorption efficiency is significantly influenced by the pore size and specific surface area of the carrier material, which may limit the quantity and stability of immobilized microorganisms [56,57]. Additionally, the connection between microorganisms and the carrier is not robust, making it prone to detachment under changing environmental conditions, thereby affecting remediation efficiency and durability.

The entrapment method involves encapsulating microorganisms within a polymer matrix or semipermeable biofilm capsules using gel-type polymers [53]. This allows small-molecule substrates and products to freely diffuse while preventing microbial escape into the external environment. The immobilized agents prepared by this method are typically spherical, exhibiting excellent cell stability and high microbial loading capacity (with live microbial content reaching 50–70%) [58]. The degradation efficiency of microorganisms is a key indicator of the success of entrapment immobilization, and this efficiency is heavily influenced by various environmental factors, particularly gel concentration, temperature, and pH [59]. Specifically, temperature directly affects the viscosity of the entrapment carrier, with high temperatures reducing gel viscosity and thereby compromising the mechanical strength of the encapsulated beads [60]. pH influences the ionic bonds between the gel and cross-linking agents, indirectly affecting the formation and mechanical strength of the beads [61]. Gel concentration, as a core determinant of gel viscosity, has a direct impact on the performance of immobilized cells. Generally, increasing gel concentration enhances the mechanical properties and density of the encapsulated beads, but excessive viscosity not only complicates the operation but also reduces pore size, hindering the transport of substrates and metabolic gases, thereby limiting microbial growth and metabolic activities [62].

Adsorption and entrapment methods, as the two mainstream approaches in microbial immobilization technology, each have unique advantages and limitations. The adsorption method is simple to operate and has minimal impact on microbial activity, but its stability and durability are limited by carrier material properties [52]. The entrapment method, on the other hand, provides higher cell stability and loading capacity through gel encapsulation, but its performance is significantly influenced by environmental factors such as gel concentration, temperature, and pH [53]. The selection between adsorption and entrapment thus involves a trade-off: adsorption offers operational simplicity and minimal impact on cell viability but suffers from lower stability [61], whereas entrapment provides superior cell retention and loading capacity at the cost of greater sensitivity to environmental parameters such as gel concentration and pH [59].

### 3.2. Carriers for Microbial Immobilization

The selection of immobilization carriers is a pivotal factor determining microbial activity, system stability, and remediation efficacy [10]. Current carrier materials are primarily categorized into inorganic carriers, organic polymer carriers, and composite carriers [63,64]. Research indicates that inorganic and organic carriers exhibit substantial differences in physicochemical properties, leading to distinct advantages and limitations in mechanical strength/durability, biocompatibility/functional tunability, and cost/environmental friendliness. Consequently, the choice of carrier is not a quest for a singular “best” option but requires careful trade-offs based on specific remediation contexts, such as the nature of pollutants, soil properties, project duration, and budget. We have summarized the performance comparison and application scenarios of typical immobilized carriers in Table 3.

Inorganic carriers generally demonstrate superior mechanical strength and chemical stability [65]. Materials such as zeolites and ceramics possess robust structures that maintain morphological integrity over long-term remediation, making them suitable for projects requiring enduring effectiveness [66,67]. However, this stability also implies they are resistant to environmental degradation; if not properly designed, they may pose legacy environmental concerns. Moreover, some high-performance inorganic materials (e.g., activated carbon with specific pore sizes) can be costly. In contrast, most organic carriers (e.g., sodium alginate gel, corncobs) exhibit lower mechanical strength and may undergo structural degradation during extended use or in complex soil environments, as observed in the study by Zhang Xiuxia et al., where the optimal organic carrier YJ-5 became porous after degradation [68]. Their advantages lie in wide availability of raw materials, low cost, and environmental friendliness. Many are derived from agricultural waste (e.g., bagasse, corncobs), enabling “waste-to-remediation” approaches [69,70].

The core strengths of organic carriers lie in their excellent biocompatibility and functional plasticity. Natural polymers like sodium alginate and chitosan contain abundant hydrophilic groups, providing a near-natural microenvironment for microorganisms and significantly preserving their metabolic activity [71,72]. More importantly, their molecular structures are easily modifiable. For instance, the study by Zhang Jingjing et al. demonstrated that modifying bagasse (MSB-Mo) markedly enhanced its degradation efficiency for mesotrione (reaching 96.35%), highlighting the flexibility in functional design of organic carriers [73]. This synergistic “carrier-microorganism” enhancement was also evident in the research by Li Haolin et al., where the activated carbon (organic) system showed significantly better purification efficacy for aquaculture water compared to inorganic carriers like zeolite [74]. Inorganic carriers, with their typically chemically inert surfaces, are difficult to functionalize. They primarily rely on physical adsorption for microbial immobilization, which can lead to uneven cell distribution, easy detachment, and consequently, compromised long-term activity.

**Table 3:** Performance Comparison and Application Scenarios of Typical Immobilization Carriers.

Carrier Type	Representative Materials	Core Advantages	Main Limitations	Key Performance Indicators	Typical Application Scenarios
Inorganic Carriers	Zeolite [66], Activated Carbon [75], Ceramics [67], Volcanic Rock [76]	High Mechanical Strength and Durability: Stable structure for long-term use; High Specific Surface Area: Strong physical adsorption capacity; Chemical Inertia: Resistant to biodegradation, low risk of secondary pollution.	Moderate Biocompatibility: Surface properties may hinder microbial attachment and growth; Poor Functional Tunability: Difficult to chemically modify for enhanced specific functions; Weak Nutrient Retention.	High-density biofilm formation: Enables 25% faster Cr(VI) removal (k = 0.0279 h^−1^) vs. free cells [76]. Superior biodiversity support: Higher OTUs (2932) and Chao index (3660.15) [66].	Long-term, high-concentration contamination; scenarios requiring robust structural support [77].
Organic Carriers	Sodium Alginate [78], Chitosan [79], Modified Bagasse [69], Biochar [80]	Excellent Biocompatibility: Hydrophilic surface favors microbial colonization; High Functional Plasticity: Easy to chemically modify with functional groups; Good Environmental Adaptability: Biodegradable, some can provide nutrients.	Lower Mechanical Strength: Prone to structural degradation during long-term use or in harsh environments; Potentially High Cost: Some synthetic polymers or modified materials can be expensive.	High cell retention: 92.3% initial retention, >85% activity after 5 cycles [69]. Mechanical protection: Compressive strength up to 65.3 mN for alginate beads, minimizing physical cell loss [78].	Short to medium-term projects; need for high microbial activity and biocompatibility; cost-sensitive applications.
Composite Carriers	Zeolite-Cornco, Biochar-Gel [80], Adsorptive Organoclay (AOC), Colloidal-Activated Carbon (CAC) [80]	Synergistic Performance: Combines structural strength of inorganic carriers with bio-affinity of organic carriers; Integrated Functionality: Enables simultaneous physical adsorption, chemical modification, and biodegradation.	Complex Preparation Process: Key challenges lie in optimizing ratios and composite techniques [81].	Synergistic habitat creation: Combines high SSA (e.g., CAC: 1352.81 m^2^/g) with organic matter to enhance microbial biomass (PLFA concentration) and resilience under redox fluctuations [80].	Treatment of complex or co-contaminated sites; environments with fluctuating conditions (e.g., redox, pH).

To bridge the performance gap of single materials, developing composite carriers has become a crucial direction. The design rationale is to combine the structural strength of inorganic materials with the bio-affinity of organic materials [82]. The research by Wu Shichun serves as a successful example: combining mechanically strong, highly adsorbent green zeolite (inorganic) with slowly carbon-releasing corncobs (organic) at a 10:1 mass ratio created a composite carrier immobilization system that significantly improved nitrogen and phosphorus removal from wastewater with a low carbon-to-nitrogen ratio (total nitrogen removal reached 98%) [81]. This demonstrates that through rational design, composite carriers can achieve a synergistic “1 + 1 > 2” effect, offering targeted solutions for complex environmental problems.

Beyond the fundamental physicochemical properties, the economic viability and reusability of carriers are decisive factors for their large-scale, sustainable application. A comparative analysis reveals distinct cost structures and recycling potentials across carrier types. Inorganic carriers, such as activated carbon or engineered zeolites, often entail significant material and processing costs. While mechanically robust, their recycling typically requires energy-intensive regeneration processes, which can offset their durability advantage in long-term cost calculations. Organic polymer carriers present a more varied economic profile. Natural polymers like sodium alginate and chitosan are relatively low-cost and amenable to simple gelation processes. More importantly, they exhibit promising reusability. For instance, sodium alginate-chitosan composite beads retained 51% product yield after 25 consecutive reaction cycles without structural failure [83], and PVA/SA/biochar carriers maintained over 95% nitrate removal efficiency through 5 reuse cycles [84]. However, the cost of some synthetic organic polymers and the potential need for frequent replacement due to biodegradation can increase long-term expenses. Composite and waste-derived carriers emerge as the most promising route for optimizing both cost and sustainability. Carriers fabricated from agricultural waste (e.g., sugarcane bagasse [69], corn straw [70], buckwheat husks, loofah sponges [85]) or industrial waste (e.g., waste polyurethane foam [86]) feature near-zero raw material costs, embodying a “waste-to-remediation” paradigm. Their preparation is often simpler and cheaper than that of synthetic carriers. Critically, these carriers demonstrate excellent reusability. For example, waste polyurethane foam showed no significant physical degradation after 14 months of continuous operation [86], and silica nanoparticle-enhanced alginate beads maintained structural integrity and residual activity over 8 reuse cycles [87]. The simple cleaning protocols required for their regeneration (e.g., rinsing with saline solution [84]) further minimize operational costs. Therefore, future carrier design must integrate performance metrics with life-cycle cost analysis. Prioritizing waste-derived or composite materials that balance high cell retention/mechanical strength (as quantified in Table 3) with low intrinsic cost and proven reusability is key to developing economically feasible and environmentally sustainable immobilization technologies for field-scale soil remediation.

In summary, the selection of an immobilization carrier requires a holistic techno-economic evaluation. It involves trade-offs not only between biocompatibility and mechanical strength but also among initial cost, long-term reusability, and environmental footprint. Future research should focus on the rational design of efficient, stable, and environmentally benign organic-inorganic hybrid carriers tailored to specific remediation contexts, thereby accelerating the translation of this technology into practical engineering applications. To accurately evaluate the performance and environmental safety of these immobilized systems, standardized assessment methods are indispensable.

### 3.3. Standardized Assessment of Microbial Remediation Efficacy

When evaluating and optimizing the degradation efficacy of microorganisms (both free and immobilized) for organic pollutants, two critical yet often underestimated dimensions are the toxic effects of contaminants and their transformation intermediates on functional microbes [88], and the standardized test methods used to scientifically assess the degradation process [89]. A deep understanding and standardized application of these aspects form the scientific cornerstone for bridging laboratory research and field-scale engineering, ensuring the environmental safety of remediation technologies.

Organic pollutants can inhibit degrading microbial consortia through various mechanisms, such as disrupting cell membranes, interfering with energy metabolism, and inhibiting the synthesis of key enzymes, directly impacting remediation efficiency. To objectively quantify this toxicity, the Organisation for Economic Cooperation and Development (OECD) and the International Organization for Standardization (ISO) have established a series of standard tests [88]. Examples include respiration inhibition tests using activated sludge (e.g., OECD 209) and nitrification inhibition tests (e.g., ISO 9509) [90]. These methods enable accurate determination of the half-maximal effective concentration (EC_50_) of pollutants, providing a basis for predicting their potential impact on microbial ecological functions in real environments (e.g., wastewater treatment plants, contaminated soil). For immobilized systems, such testing helps predefine safe operational concentration ranges for target contaminants, preventing the inactivation of the carrier-entrapped consortium due to toxic shock, thereby supplying crucial parameters for the robust design of immobilized agents. The tiered testing framework under the OECD/ISO guidelines is globally recognized as the “gold standard” for assessing whether a pollutant “can be degraded by microorganisms” and “how readily” [88,89]. This framework typically consists of three levels: (1) Ready biodegradability tests (e.g., OECD 301 series [91]), which screen for highly degradable substances under stringent conditions; (2) Inherent biodegradability tests (e.g., OECD 302B [92]), which evaluate the potential degradability of substances under more favorable conditions; and (3) Simulation tests (e.g., OECD 308, 309 [93]), which assess degradation behavior and fate in systems simulating specific environments like soil or surface water. In recent years, comprehensive metrics such as the Physiological Potential of the Inoculum (PPI) have been proposed to simultaneously characterize the biodegradation adaptation potential (BAP) and chemical resistance potential (CRP) of microbial communities [88]. This provides a more scientific theoretical framework for screening and constructing efficient, stable immobilized microbial consortia. In practical application and process monitoring, rapid biotoxicity test systems like Microtox^®^ [94], based on luminescent bacteria, have demonstrated unique value. Their principle is to rapidly reflect comprehensive toxicity by measuring the inhibition of luminescence in *Vibrio fischeri* by pollutants [95]. Studies have confirmed that this system can be used to monitor the dynamic changes in the toxicity of environmental matrices (e.g., water, soil elutriates) during remediation in real time [96]. Its Toxicity Unit (TU) shows significant correlation with conventional indicators like Chemical Oxygen Demand (COD). Therefore, it serves not only as an effective tool for assessing remediation endpoints but also provides immediate feedback for the dynamic optimization and adjustment of the remediation process.

In summary, integrating standardized toxicity and biodegradability assessments into the development and evaluation pipeline of microbial remediation technologies particularly immobilization strategies is a critical step towards transitioning these technologies from the laboratory to the field, and from mere efficiency to predictability and environmental safety. Future research should place greater emphasis on utilizing these standardized tools to scientifically elucidate how immobilization carriers mitigate toxicity stress and optimize consortium functionality, thereby systematically enhancing the reliability and applicability of the technology in complex real-world environments.

### 3.4. Efficiency of Immobilized Microbial Communities and Influencing Factors

The efficiency of microbial remediation of organic pollution is influenced by various environmental factors, including initial pollutant concentration, carrier dosage, and carrier physical properties [97]. Nuhoglu et al. investigated the effects of different initial phenol concentrations (110–420 mg/L) on microbial growth and degradation efficiency. In simulated wastewater, the microbial growth curve exhibited a typical “S” shape, including lag, logarithmic, and stationary phases [98]. As the phenol concentration increased, the growth rate during the logarithmic phase gradually decreased, indicating that high phenol concentrations inhibited microbial growth. However, phenol acts as both a toxin and a nutrient. At 110 mg/L, the microbial growth rate was the fastest, but the microbial concentration was lower than in systems with 220 mg/L and 420 mg/L, suggesting insufficient nutrient supply at this concentration. At 420 mg/L, the microbial concentration did not exceed that of the 220 mg/L system, indicating that the microorganisms had reached their maximum growth limit. Microbial growth showed strong temporal consistency with phenol concentration reduction, with the fastest phenol degradation occurring during the logarithmic phase, while phenol concentration ceased to decrease after the stationary phase. Except for complete degradation at 110 mg/L, phenol at 220 mg/L and 420 mg/L was not fully degraded, with higher concentrations resulting in greater residual amounts. Although high phenol concentrations provided sufficient metabolic substrates, their increased toxicity ultimately inhibited further microbial utilization. Therefore, the efficiency of microbial remediation is closely related to the initial concentration of pollutants.

The properties of the carrier also significantly influence pollutant removal efficiency. Chen et al. studied the effects of biochar prepared at different temperatures on phenol degradation. The results showed that the specific surface area of biochar varied significantly with preparation temperature [99]. The specific surface area of biochar increased significantly with higher temperatures, facilitating pollutant adsorption and microbial attachment, making it an excellent carrier for microbial growth. Additionally, differences in surface functional groups of biochar led to variations in microbial adsorption capacity, thereby affecting pollutant distribution and ultimately resulting in differences in phenol degradation efficiency. When the environmental pollutant concentration increased, different immobilization carriers exhibited significant differences in microbial adsorption capacity. Gong et al. conducted a systematic comparison of organic and inorganic materials as immobilization carriers for a mixed consortium of PAH-degrading microorganisms under low-temperature conditions [99]. After 60 days of soil remediation experiments, vermiculite-based inorganic carriers demonstrated superior PAH degradation efficiency compared to other tested matrices. Quantitative analysis revealed enhancement in degradation rates of phenanthrene, pyrene, and benzo[a]pyrene by 29.09%, 11.56%, and 12.78%, respectively, over free-cell systems, which may be attributed to the carrier’s specific surface area and charge characteristics.

Although inorganic carriers (e.g., vermiculite, zeolite) exhibit excellent remediation performance under controlled laboratory conditions with notable advantages including environmental friendliness (non-toxicity), high mechanical strength, cost-effectiveness, and chemical stability [100], their practical application in field-scale remediation faces significant challenges. The primary limitation stems from the limited variety of surface functional groups on inorganic carriers, resulting in insufficient interfacial affinity with microbial cells. This often leads to carrier–microbe dissociation in complex soil matrices, ultimately compromising immobilization efficacy. In contrast, organic carrier materials (e.g., agricultural waste, biochar) have garnered increasing attention due to their multifaceted advantages [101]: (1) The abundance of surface functional groups (e.g., carboxyl, hydroxyl) significantly enhances microbial adhesion stability; (2) Their wide availability as renewable resources coupled with simple preparation processes offers distinct economic and environmental benefits; (3) Most importantly, these materials not only effectively immobilize functional microorganisms but also synergistically enhance remediation through soil structure improvement and nutrient supplementation. These combined characteristics make organic carriers particularly promising for practical applications in organically contaminated site remediation.

Therefore, the remediation efficiency is not determined by a single factor but by the interplay of initial pollutant concentration, carrier physicochemical properties (e.g., specific surface area, functional groups), and ambient environmental conditions. Optimizing these parameters in concert is crucial for enhancing microbial tolerance and degradation performance.

### 3.5. Functional Mechanisms of Microbial Immobilization Technology in Soil Remediation

In the field of soil remediation, the core principle of immobilized microbial communities lies in their biological metabolic activities, which encompass adsorption, precipitation, redox reactions, and complexation mechanisms [102]. The synergistic effects of these mechanisms aim to reduce the bioavailability of pollutants in soil, thereby achieving soil remediation. Specifically, immobilized microbial communities utilize various functional groups on their cell surfaces (e.g., carboxyl, hydroxyl, and amino groups) to bind with pollutants, forming stable complexes that effectively reduce the biological activity of pollutants [103]. Simultaneously, these microbial communities convert soluble pollutants into insoluble forms or transform highly toxic substances into less toxic forms through redox reactions, methylation and demethylation processes, dissolution, and organic complexation degradation [80,104]. Furthermore, specific metabolites produced by microbial communities can combine with pollutants to form precipitates, significantly reducing the mobility and bioavailability of pollutants in soil [105]. Table 4 provides a detailed summary of the types of microbial immobilization carriers and their applications in soil remediation.

Biochar is a common carrier material for microbial immobilization, and its structural and property complexity results in diverse and intricate mechanisms for organic pollutant degradation [106]. Firstly, the porous nature of biochar enables adsorption of organic pollutants, which may positively promote degradation or hinder microbial utilization by “locking” pollutants on the biochar surface or within its pores. Chen and Yuan found that pinewood biochar, as a carrier for *Pseudomonas putida*, exhibited strong adsorption affinity for polycyclic aromatic hydrocarbons (PAHs), accelerating degradation by shortening the contact distance between pollutants and microorganisms [99]. Oh and Seo evaluated the adsorption of several halogenated phenols (including 2-chlorophenol, 4-chlorophenol, and 2,4-dichlorophenol) on biochar, revealing adsorption capacities ranging from 24.6 to 47.7 mg/g [107]. The pore structure and surface properties of biochar significantly influence its adsorption capacity and mass transfer efficiency for organic pollutants.

Secondly, biochar typically exhibits alkalinity due to its deprotonated surface functional groups and carbonate content, with pH increasing as pyrolysis temperature rises, reaching up to 11 at 700 °C. This characteristic affects the environmental pH in aqueous or soil phases, resulting in dual effects: altering the molecular forms of organic pollutants and regulating microbial growth, thereby influencing degradation processes. Pietikainen et al. studied the treatment of simulated wastewater contaminated with 430 mg/L phenol by coculturing microorganisms with 0.2%, 0.4%, and 0.6% (*w*/*v*) peanut shell biochar pyrolyzed at 550 °C, observing phenol degradation and pH changes [108]. The degradation efficiency of phenol by microorganisms alone was only 36.3%, while the addition of biochar significantly enhanced phenol removal, primarily through adsorption in the initial 6 h, with reductions of 12.0–39.3%, positively correlated with biochar dosage. Subsequently, microorganisms entered the logarithmic growth phase, and phenol concentrations continued to decline sharply, with complete removal achieved within 16 h. In contrast, the standalone microbial system, inhibited by phenol toxicity, exhibited slow growth and a maximum degradation rate of only 38.2% at 16–25 h. Biochar not only reduced phenol concentration through adsorption but also promoted microbial growth. Additionally, the alkalinity of biochar increased the culture solution pH, with a maximum of 7.58. The initial pH was adjusted to 7.0 using hydrochloric acid to favor microbial growth. At the end of the experiment, the pH of the free microbial group dropped to 4.27, while the 0.2% biochar group dropped to 3.74, likely due to the production of acidic intermediates from complete phenol degradation, with insufficient biochar to neutralize the acidity. As biochar content increased, the solution pH gradually rose. Furthermore, water-soluble organic components released during high-temperature pyrolysis of biochar, such as small molecular acids and polycyclic aromatic hydrocarbons, may influence the biodegradation of organic pollutants. These components can serve as valuable carbon sources for microbial growth or inhibit microbial activity [109].

Yamasaki H et al. investigated the degradation efficiency of PYR by free strain BQ-3, hydrogel microspheres without bacteria, and hydrogel microspheres immobilized with strain BQ-3 [54]. The results showed that the immobilized hydrogel microspheres exhibited higher PYR degradation efficiency than free strain BQ-3, indicating that the introduction of immobilization carriers enhanced PYR degradation performance. By encapsulating free strains within a dual-crosslinked network structure composed of ALG/CMC/Zeolite P, the utilization efficiency of PYR by the strains was significantly improved. Wang et al. used immobilized *Candida tropicalis* to remediate PYR-contaminated soil and found that the degradation rate of immobilized strains was 20% higher than that of free cells [110]. The improved degradation efficiency was attributed to the rich pores within the hydrogel microspheres, which facilitated high-density bacterial attachment, while the microspheres provided a protective barrier against external environmental disturbances [111]. The high specific surface area of the hydrogel microspheres likely promoted pollutant absorption, increasing the internal PYR concentration and enhancing contact opportunities between pollutants and microorganisms, thereby improving biodegradation efficiency.

**Table 4:** Types of microbial immobilization carriers and their applications in soil remediation.

Immobilization Carriers	Microbial Species	Immobilization Techniques	Target Contaminant	Degradation Rate (%)	Treatment Duration (d)	Degradation Efficiency Improvement (%)	References
Coal slag-derived biochar	*Pseudomonas putida* PYR1 and *Acinetobacter baumannii* INP1	Embedding	Pyrene/indeno(1,2,3-cd)pyrene	70.7/80.9	30 d	12.5/25.6	[112]
Pine needle-based biochar (600 °C)	*Sphingomonas* sp. PJ2	Adsorption	TPHs	58.64	60 d	37.3	[113]
Corn stalk biochar	*Vibrio* sp. LQ2	Adsorption	Diesel oil	94.7	7 d	40.3	[114]
Wheat bran-based biochar	*Pseudomonas*, *Acinetobacter*, and *Sphingomonas* for PAHs degradation	Adsorption	TPHs	58.31	84 d	14.33	[115]
Rice husk stover biochar	*Mycobacteria M. gilvum*	Adsorption	Fei/Pyrene	62.6 ± 3.2/ 62.1 ± 0.9	18 d	15.3/42.4	[116]
Plant residue composite biochar	*Pseudomonas putida*	Embedding	Fei/Pyrene	77.2/72.4	21 d	65.2/60.4	[99]
Hydroxyethyl cellulose/luffa composite sponge	*Bacillus thuringiensis*, *Pseudomonas aeruginosa*, *Acinetobacter lwoffii*, *Nocardioides luteus*, *Penicillium oxalicum*	Embedding	Oils	94.50	7.5 d	72.8	[61]
Coconut coir biochar	*Paenarthrobacter* spp., *Zoogloea* spp.	Adsorption	Para-nitrophenol	99	2.5 d	89	[117]
Modifed wheat straw biochar	Firmicutes and Proteobacteria	Adsorption	Benzo[α]pyrene (BαP)	75.18	12 d	18.68	[118]
Kapok fibers biochar	Petroleum hydrocarbon-degrading bacteria	Cross-linking	Diesel oil and emulsified diesel oil	94.98	8.5 d	45	[119]
Corn straw biochar	*Klebsiella jilinsis*	Adsorption	Nicosulfuron	93.38	4 d	0.76	[120]
Biochar derived from the stem of *Solidago canadensis* L.	*Stenotrophomonas maltophilia* J2	Adsorption	Pyridine	98.66 ± 0.47	32 d	67.44	[121]

The functional mechanisms of microbial immobilization technology in soil remediation primarily rely on the synergistic effects of microbial metabolic activities and carrier materials. Through mechanisms such as adsorption, precipitation, redox reactions, and complexation, immobilized microorganisms effectively reduce the bioavailability of pollutants and transform them into less toxic or insoluble forms [122]. Carrier materials like biochar and hydrogel not only promote pollutant degradation through physical adsorption and chemical interactions but also provide a suitable growth environment for microorganisms, enhancing their tolerance and degradation efficiency [109]. Therefore, advancing the field requires moving from phenomenological observation to mechanistic prediction. Future research must prioritize deploying advanced in situ imaging and analytical tools such as nano-scale secondary ion mass spectrometry (nano-SIMS) and confocal laser scanning microscopy coupled with metabolic flux analysis to spatially resolve and quantify the critical interfacial processes (e.g., electron transfer, substrate diffusion) within defined immobilization matrices, such as the alginate-based hydrogels studied by Yamasaki et al. [54] and Wang et al. [110]. By correlating specific carrier architectural features (e.g., pore size distribution, functional group density) with these quantified microbial processes, we can establish design principles for next-generation carriers that actively manage the micro-environment to optimize degradation kinetics and cell longevity.

### 3.6. Practical Applications of Microbial Immobilization Technology in Soil Remediation

Microbial immobilization technology has achieved remarkable success in soil remediation, demonstrating superior performance in various contamination scenarios [102]. Huang et al. immobilized two polycyclic aromatic hydrocarbon (PAH)-degrading strains on coal slag carriers for enhanced microbial remediation of wetland soil. After a 30-day remediation period, the degradation efficiency of pyrene increased by 12.2% compared to non-immobilized free cells [112]. Song et al. immobilized a PAH-degrading strain, *Sphingomonas* sp. PJ2, isolated from a coking plant, on pine needle biochar for the remediation of farmland soil near the Liaohe oil-contaminated site [113]. After 60 days of treatment, the total PAH degradation rate of the immobilized agent was 15.94% and 37.3% higher than that of the carrier alone and free cells, respectively, demonstrating significant remediation efficiency.

Guan Rui et al. investigated the mechanism of di(2-ethylhexyl) phthalate (DEHP) degradation through the synergistic action of Arthrobacter sp. strain JQ-1 and corn straw biochar (BC) at 600 °C, and conducted bacterial viability, microenvironmental alteration, and kinetic tests. Compared to the sole biodegradation group, the degradation rates were enhanced by 18.3% and 30.9%, respectively. The study demonstrated that biochar (1% (*w*/*v*)) utilizing JQ-1 as a DEHP-removing material exhibited favorable performance [123]. Zhou et al. employed corn stalk biochar as a carrier to immobilize *Vibrio* sp. LQ2, a biosurfactant-producing strain, for the treatment of marine diesel contamination. The results showed that the residual diesel content decreased significantly from 169.2 mg to 8.91 mg, with a degradation rate of 94.7%, outperforming the treatments using biochar alone (35.2% degradation rate) and free cells (54.4% degradation rate) [114].

Zhou et al. screened and domesticated a native synthetic microbial consortium J3 with high efficiency in polycyclic aromatic hydrocarbon (PAH) degradation from coking plant–soil, and prepared immobilized microbial consortium using corn straw as a carrier for the remediation of PAH-contaminated soil. After 20 days of cultivation, the application of 5% JLS (immobilized microbial consortium) increased the removal rate of PAHs in the soil to 88%, demonstrating that the immobilization of native bacterial consortia using crop straw as a carrier material is a more efficient bioremediation technology for degrading PAHs in contaminated soil [70]. Lu et al. immobilized *Pseudomonas aeruginosa* using a composite of rice straw biochar (RS500) and alginate through gel entrapment technology to treat acenaphthene in water at a concentration of 3.5 mg·L^−1^. The results indicated that the addition of the nonionic surfactant TX100 effectively enhanced the adsorption of PAHs by biochar and improved the degradation capacity of *Pseudomonas aeruginosa*. Under optimal conditions, the degradation rate of acenaphthene by *Pseudomonas aeruginosa* immobilized in alginate-biochar reached 50.6% [124].

The practical applications of microbial immobilization technology in soil remediation have fully demonstrated its high efficiency and broad applicability. By immobilizing functional strains on carriers such as biochar and coal slag, the degradation efficiency of pollutants has been significantly improved, and the adaptability of microorganisms to complex contaminated environments has been enhanced [125]. The promising results from these pilot-scale applications, however, highlight a significant translational gap. Data on long-term (e.g., exceeding two years) remediation efficacy, ecological side effects (e.g., on soil microbiota succession and nutrient cycling), and economic performance at full field-scale are critically lacking. Bridging this gap requires dedicated long-term monitoring studies and robust techno-economic analyses under real-world conditions.

## 4. Challenges of Microbial Immobilization Technology

### 4.1. Enhancing Microbial Community Stability and Adaptability

Improving the stability and adaptability of microbial communities is a major bottleneck in soil remediation using immobilized microorganisms [126]. Optimizing external environmental conditions is fundamental to maintaining microbial community stability, with precise control of temperature and pH being crucial to ensure optimal growth and reproduction [50]. Additionally, providing sufficient and balanced nutrients, adjusted according to microbial metabolic needs and growth stages, is essential. In terms of microbial community management, regular monitoring and evaluation of the composition, structure, and functional characteristics of microbial communities are necessary to identify and address potential issues promptly [127].

Adaptability refers to the ability of microbial species to adjust to specific environmental conditions, maintaining high activity and degradation efficiency under varying circumstances [117]. Screening for stress-resistant microorganisms (e.g., acid-resistant, alkali-resistant, and heat-resistant strains) can improve community adaptability in harsh environments [128]. Adaptive laboratory evolution is often used to induce mutations and select for desirable traits, with microorganisms evolving under artificial selective pressure, and individuals with superior characteristics being selected from the evolved population [129]. Additionally, genetic engineering can be employed to modify microorganisms, enhancing their environmental adaptability and degradation capabilities [20]. However, the environmental release of genetically modified microorganisms (GMMs) raises important biosafety concerns, including the potential for horizontal gene transfer to indigenous microbiota, unintended ecological impacts, and persistence beyond the in-tended remediation timeframe [130]. Consequently, the application of GMMs is strictly regulated. Current research is therefore also advancing contained use strategies such as the incorporation of biological containment circuits (e.g., kill switches, nutrient auxo-trophy) to mitigate potential environmental risks while harnessing the enhanced functionality of engineered strains. Studying microbial interactions (e.g., symbiosis, competition, and antagonism) and understanding how microorganisms adapt to environmental changes can help construct more stable and efficient microbial communities [131].

In constructing immobilized microbial systems for soil remediation, the scientific selection of inoculants is a critical preliminary step to ensure community stability and adaptability. The Physiological Potential Index (PPI) provides a structured, quantitative framework for this purpose [88]. As a core metric for evaluating inoculant quality and functional compatibility, PPI assesses microbial potential for degrading soil organic pollutants through two integrated dimensions: Biodegradation Adaptation Potential (BAP) and Chemical Resistance Potential (CRP). BAP is graded based on the lag phase duration during pollutant degradation, with shorter lag periods indicating stronger adaptive and induction capacity for recalcitrant compounds [132]. CRP is classified according to EC_50_ values derived from standardized toxicity assays, where higher tolerance thresholds reflect greater resilience to co-occurring contaminants [133]. By combining BAP and CRP scores, PPI enables tiered inoculant selection: high-PPI consortia (high BAP + high CRP) are suited for heavily contaminated sites with complex pollutant mixtures; medium-PPI inoculants match moderate organic pollution scenarios; and low-PPI strains are generally unsuitable for challenging remediation contexts. Furthermore, PPI assessment should account for inoculant provenance sludges acclimated to industrial contamination typically exhibit higher PPI than those from municipal wastewater treatment plants [134]. This systematic approach minimizes the risk of remediation failure due to inappropriate inoculant choice and provides a scientific basis for building stable, adaptable immobilized microbial systems.

In summary, advancing the stability and adaptability of microbial communities remains central to the effectiveness of immobilization-based soil remediation. Through integrated strategies including environmental conditioning, stress-tolerant strain selection, responsibly leveraging genetic engineering within appropriate regulatory and biosafety frameworks, and interaction-informed consortium design we can substantially enhance community performance. Incorporating quantitative selection tools such as the Physiological Potential Index further strengthens this foundation by ensuring that inoculants are matched to pollutant profiles and environmental stresses from the outset, thereby improving both the reliability and efficiency of the remediation process.

### 4.2. Impact of Environmental Factors on Microbial Community Stability and Mitigation Strategies

Environmental factors have multidimensional effects on microbial community stability, directly or indirectly influencing microbial structure, function, and overall stability [135]. Temperature is a critical factor, as excessively high or low temperatures can disrupt microbial community balance. Environmental pH also significantly impacts microbial growth and metabolism, with overly acidic or alkaline conditions threatening community stability. Additionally, the type and concentration of nutrients directly affect microbial growth and reproduction. Insufficient essential nutrients can alter microbial community structure and function, while nutrient excess may lead to overgrowth of specific microorganisms, disrupting community balance.

Oxygen concentration significantly influences the growth of anaerobic and aerobic microorganisms. Hypoxic conditions may favor anaerobic microorganisms, while oxygen-rich environments may promote aerobic microorganisms, both of which can alter community structure [136]. Water, as an essential requirement for microbial growth and metabolism, can negatively impact microbial communities if deficient or excessive, threatening community stability [137]. Environmental pollution, including air, water, and soil contamination, can adversely affect microbial communities. For example, heavy metal pollution may cause DNA damage and reduced enzyme activity [138], while chemical pollutants and microplastics can alter microbial growth environments and metabolic pathways [139]. Additionally, strong radiation can directly kill microorganisms or cause genetic mutations, threatening community stability and diversity [140].

To maintain microbial community stability, a series of comprehensive measures must be implemented. First, optimizing culture conditions based on microbial growth requirements and metabolic characteristics, including precise control of temperature, pH, and provision of balanced nutrients, is essential to create a suitable environment for microbial growth [127]. Therefore, strengthening environmental monitoring is crucial. Regular monitoring of key indicators such as temperature, pH, oxygen concentration, and pollutant levels can help identify and address potential environmental issues promptly. Additionally, advanced biotechnological approaches such as genetic engineering and metabolic engineering can be used to modify and optimize microorganisms, significantly enhancing their adaptability and tolerance to environmental factors [20]. Finally, establishing a microbial community stability assessment system allows for regular evaluation and analysis of community stability, enabling timely identification and resolution of stability issues to ensure long-term microbial community stability [117]. Consequently, bridging the lab-to-field gap requires proactive strategies to engineer ecological resilience into immobilized consortia. Future efforts should focus on: (i) Pre-adaptation through simulated soil microcosms that expose consortia to dynamic gradients of key stressors (e.g., oscillating oxygen [136] and moisture levels [137]) to select for robust genotypes; (ii) Rational consortium design that pairs degraders with stress-protecting partners or incorporates protective amendments (e.g., biochar to sequester heavy metals [138] and mitigate toxicity) within the carrier; and (iii) Employing ‘eco-mimetic’ carriers derived from natural materials (e.g., corn straw [70]) that may facilitate more seamless integration with the soil habitat and native microbial networks. The goal is to shift from introducing vulnerable ‘foreign’ agents to deploying self-sustaining, ecologically competent remediation units.

### 4.3. Challenges in Scaling Up Immobilized Microbial Technology

Scaling up immobilized microbial technology from laboratory to field applications faces several intertwined technical and economic challenges. One of the primary technical bottlenecks lies in the economic viability and long-term durability of immobilization carriers. Specifically, the cost of carriers constitutes a significant portion of the overall treatment expenditure [141]. A preliminary techno-economic analysis based on recent pilot studies indicates that carrier materials themselves can account for 20% to 60% of the total project cost, depending on the material source (e.g., commercial activated carbon vs. agricultural waste-derived biochar) and the required processing level. Concurrently, the operational lifespan of these carriers directly dictates the frequency of re-application and thus impacts the long-term stability and cost-effectiveness of the remediation system [135]. Beyond carrier costs, the economic feasibility is further influenced by several key parameters: Immobilization process costs, including energy inputs for sterilization, mixing, and drying; Nutrient and maintenance costs required to sustain microbial activity in situ; and monitoring and validation costs associated with tracking remediation progress and ensuring compliance.

In practical applications, such as wastewater treatment, immobilized microbial cells may also encounter operational issues like carrier swelling or clogging when handling specific suspended solids or polymers, which can increase maintenance costs and reduce system longevity [142]. Furthermore, the activity of immobilized cells is influenced by multiple interacting factors, necessitating the exploration of optimal operating parameters to maximize treatment outcomes and economic returns [143]. Another critical challenge lies in ensuring the efficient release and desorption of microorganisms in certain applications. For instance, in soil remediation, the release efficiency of immobilized cells directly governs their contact with pollutants, thereby influencing the overall remediation kinetics and cost-effectiveness [144]. Additionally, scaling up requires careful consideration of the recyclability and environmental friendliness of carrier materials to minimize secondary pollution and operational costs [145].

In summary, the path to large-scale implementation is not merely a technical scaling exercise but an economic optimization challenge. Future development must prioritize the design of low-cost, high-durability carriers (e.g., from waste streams), the standardization of efficient immobilization protocols to reduce processing expenses, and the establishment of comprehensive life-cycle cost models that integrate initial investment with long-term performance and maintenance data. Demonstrating clear cost advantages over conventional remediation methods through robust field-scale case studies will be pivotal for industry acceptance and regulatory endorsement.

## 5. Conclusions

Microbial immobilization technology has evolved from a laboratory concept into a sophisticated bioremediation strategy aimed at overcoming the limitations of free microbial cells. This review synthesizes current advances and establishes that the efficacy of this technology is governed by an integrated Carrier–Consortium–Context (3C) framework.

First, the Carrier serves as more than a passive support; it is an active component that modulates the microenvironment. The choice between organic (e.g., alginate, biochar) and inorganic (e.g., zeolite) materials involves a critical trade-off between biocompatibility/cost and mechanical strength/durability, necessitating context-specific design.

Second, the Consortium must be ecologically resilient, not merely metabolically potent. While lab studies show degradation rate improvements of 20–60%, field success depends on the consortium’s ability to compete with indigenous microbes and withstand fluctuating stresses (e.g., O_2_, moisture). Future strategies must prioritize selecting and engineering consortia for in situ fitness, using tools like the Physiological Potential Index (PPI) and pre-adaptation to environmental gradients.

Third, successful field-scale Context integration demands moving beyond pure performance metrics. The high cost of carriers and uncertain long-term ecological impacts remain key bottlenecks. Sustainable scale-up requires (i) developing low-cost, waste-derived carriers (e.g., agricultural residues); (ii) establishing standardized lifecycle assessments that combine techno-economic analysis with ecological monitoring; and (iii) validating these systems through robust, long-term (>2 years) field trials.

In essence, the path forward lies in the convergence of materials science, microbial ecology, and process engineering. By adopting the integrated 3C framework, research can shift from producing isolated solutions to developing predictable, robust, and economically viable platforms for the sustainable remediation of organically contaminated soils.

## Figures and Tables

**Table 1:** Overview and comparative analysis of remediation technologies for organic contaminated soils.

Remediation Technologies	Technologies/Methods	Advantages	Limitations	References
Physical Remediation Technologies	Soil Replacement Vitrification Remediation Thermal Treatment Electrothermal Desorption Thermal Conduction Heating Steam-Enhanced Extraction	Rapid effectiveness; short treatment duration; excellent stability.	Temporary containment rather than permanent solution; contaminant relocation rather than destruction; failure to address the fundamental pollution source	[18]
Chemical Remediation Technologies	Chemical Oxidation Plasma Degradation Photocatalytic Degradation	High contaminant removal rates; rapid chemical reaction kinetics; significant reduction in treatment duration; minimal influence from soil environmental factors	Introduction of new chemical substances; potential for secondary pollution events; disruption of native soil microbial communities; alteration of soil chemical properties; possible long-term ecological consequences	[19]
Biological Remediation Technologies	Microbial Remediation Phytoremediation Zooremediation	Low implementation costs; reduced need for expensive equipment; sustainable and eco-friendly approach; complete mineralization capability; generation of non-toxic end-products; preservation of soil structure and function	Slow remediation rate; low treatment efficiency; extended process duration; high sensitivity to environmental factors; sensitivity to contaminant bioavailability	[20]

**Table 2:** Classification, mechanisms, and comparative analysis of microbial immobilization techniques.

Method	Principle	Advantages	Limitations	References
Adsorption	Utilizes microbial ability to adhere to solid surfaces; employs natural adhesion mechanisms (physical adsorption, ionic bonding, electrostatic attraction); forms biofilms on insoluble carriers or porous media	Simple operation procedures; mild reaction conditions; minimal impact on microbial viability; reusability of carrier materials; well-established traditional method	Weak microbial–carrier interactions; limited binding stability; carrier-dependent microbial loading capacity; restricted surface area utilization; restricted microbial density; potential for performance inconsistency	[52]
Embedding	Utilizes gel or polymer matrices to encapsulate microbial cells; creates semi-permeable membranes or network structures; allows diffusion of small molecules while retaining microbial cells; forms immobilized cells within porous carriers	Simple implementation process; minimal impact on microbial viability; high cell retention and stability; large microbial loading capacity; preservation of multi-enzyme systems; protection against environmental stress; stable operation parameters; versatile matrix options;	Process sensitivity to environmental factors; potential mass transfer limitations; material-dependent molecular size selectivity; optimization requirements for specific applications; potential oxygen diffusion constraints; sensitivity to gel concentration	[53]
Cross-linking	Utilizes multi-functional cross-linking agents; forms covalent bonds with cell surface groups; creates stable intermolecular connections; establishes robust immobilized structures	strong intermolecular bonding; resistance to environmental changes; durable immobilization matrix; stable network architecture; environmental stress resistance; temperature and pH tolerance; long-term stability	Harsh chemical reaction conditions; potential cellular activity reduction; possible enzyme activity loss; risk of cellular damage; process sensitivity optimization	[54]
Covalent bonding	Forms strong chemical bonds between cells and surfaces; utilizes functional groups on cell surfaces; creates stable, permanent attachments	Excellent stability; firm attachment to support surface; long-lasting microbial fixation; robust chemical linkages; strong surface bonding; long-term durability	Harsh reaction conditions; complex operational procedures; difficult process control; high cell mortality rate; parameter optimization needs; reaction condition sensitivity	[55]

## Data Availability

No new data were created or analyzed in this study. Data sharing is not applicable to this article.
